# Supplementary material for: High-order species interactions shape ecosystem diversity
Source: Nat Commun. 2016 Aug 2;7:12285. doi: 10.1038/ncomms12285 (PMC4974637; doi:10.1038/ncomms12285)
Supplement: Supplementary Information — Supplementary Figures 1-9 [file ncomms12285-s1.pdf]

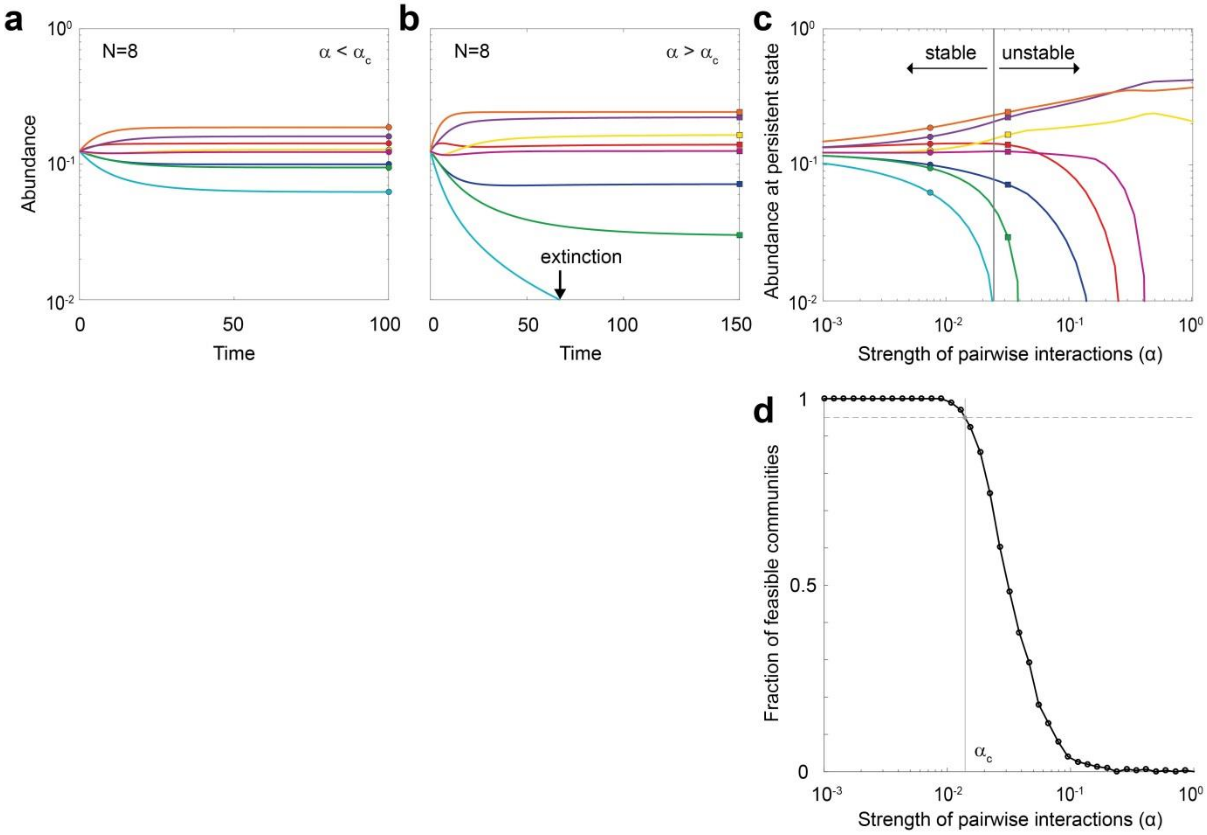

Supplementary Figure 1: **Determining the critical intensity of interactions. a-b,** Example simulations of *N*=8 species communities with the same random pairwise interactions matrix $\tilde{A}$ scaled by interaction strengths below and above the critical strength. The weakly interacting community in panel (a) converges to a stable fixed-point with all species coexisting, while the strongly interacting community in panel (b) exhibits a species extinction. **c,** The final species abundances at the end of the simulation for communities with the same matrix $\tilde{A}$ from panels (a-b) scaled by increasing interaction strengths α. Circles and squares correspond to final abundances in panels (a) and (b) respectively. The communities lose their feasibility when interactions are increased to the point where one or more of the species becomes extinct. **d,** This process is repeated for different matrices $\tilde{A}$, and the critical interaction strength α_c is defined as the strength where the fraction of feasible communities drops to 95%.

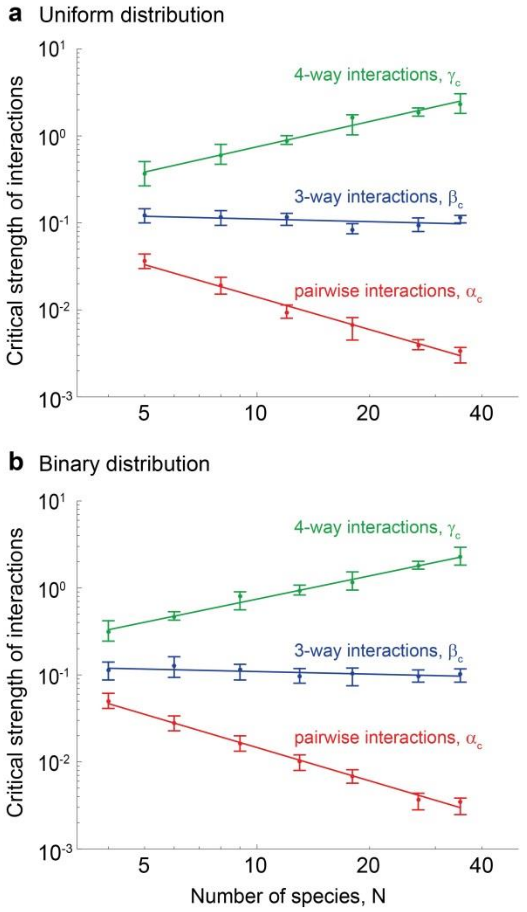

Supplementary Figure 2: Results are robust to changing the distribution of the interaction coefficients. Procedure from Fig. 2 was repeated, but instead of drawing the elements of the random matrices $\tilde{A}, \tilde{B}, \tilde{C}$ from a Gaussian distribution, different distributions with mean 0 and variance 1 were used, and the critical value of interactions of different orders at which 5% of random communities become unfeasible was plotted against the number of initial species (error-bars indicate the range of 2-10%). **a,** Uniform distribution in the interval $[-\sqrt{3}, \sqrt{3}]$. Slopes for $\alpha_c, \beta_c, \gamma_c$ respectively: $-1.24 \pm 0.21, -0.10 \pm 0.25, 0.97 \pm 0.20$. **b,** Binary distribution taking the values ± 1 in equal probability. Slopes for $\alpha_c, \beta_c, \gamma_c$ respectively: $-1.27 \pm 0.12, -0.10 \pm 0.10, 0.89 \pm 0.12$.

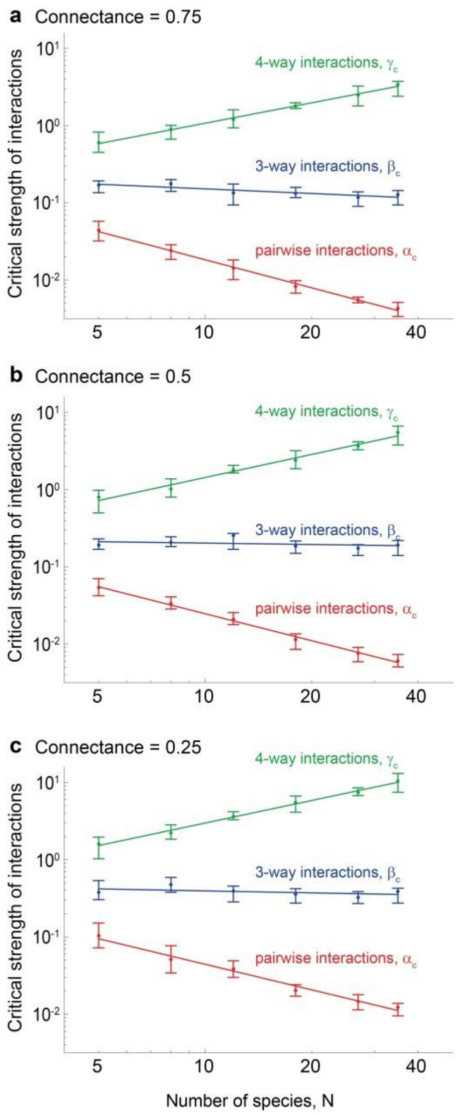

Supplementary Figure 3: Results are robust to changing the connectance of the interaction matrices. Procedure from Fig. 2 was repeated, but instead of assuming full interaction networks, we looked at sparse interaction matrices $\tilde{A}, \tilde{B}, \tilde{C}$, where the probability of each element being non-zero is set to *C*. Generalizing the notion of connectance to 3-way interactions, we defined it as the probability that species *j,k* have a joint effect upon species *i*, therefore the elements $\tilde{B}_{\mathrm{ijk}}, \tilde{B}_{\mathrm{ikj}}$ were either both zero or both non-zero, and similarly with the 4-way interaction elements. Critical interaction strengths are plotted against number of initial species for: **a,** *C*=0.75. Slopes for $\alpha_c, \beta_c, \gamma_c$ respectively: $-1.21 \pm 0.1, -0.20 \pm 0.14, 0.88 \pm 0.07$. **b,** *C*=0. 5. Slopes for $\alpha_c, \beta_c, \gamma_c$ respectively: $-1.16 \pm 0.11, -0.06 \pm 0.23, 0.99 \pm 0.18$. **c,** *C*=0.25. Slopes for $\alpha_c, \beta_c, \gamma_c$ respectively: $-1.11 \pm 0.19, -0.08 \pm 0.20, 0.97 \pm 0.10$.

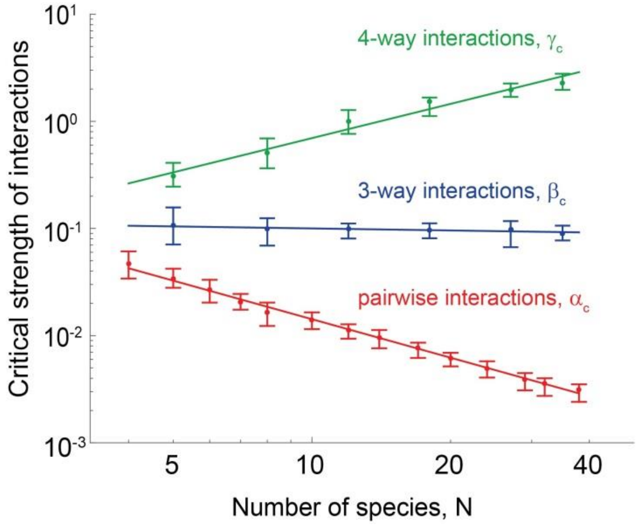

Supplementary Figure 4: Results are robust to turning off high-order diagonal coefficients. Procedure from Fig. 2 was repeated with 3-way interactions involving strictly 3 different species. We turned off elements with two or more identical indices, which may be interpreted as non-linear pairwise interactions or self-limitation (such as $\widetilde{B}_{\mathrm{ijj}}$ or $\widetilde{B}_{\mathrm{iii}}$). To avoid redundancy, elements $\widetilde{B}_{\mathrm{ijk}}$ with *j*>*k* were also turned off, as $\widetilde{B}_{\mathrm{ijk}}, \widetilde{B}_{\mathrm{ikj}}$ both represent the joint effect of species *j*, *k* on species *i*. To account for the missing elements, the critical strength of interactions was normalized by dividing it by the proportion of non-zero elements (blue, slope = -0.06 ± 0.05). The same scheme was repeated for 4-way interactions (green, slope = 1.06 ± 0.24); results for pairwise interactions (red) are taken from Fig. 2.

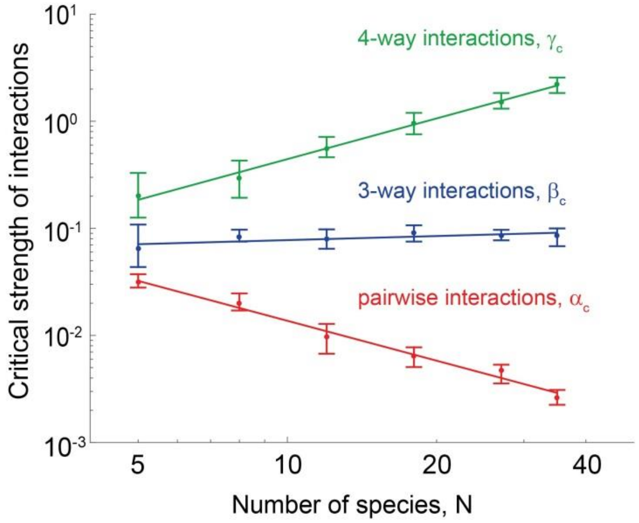

Supplementary Figure 5: Results hold for a Lotka-Volterra model. Procedure from Fig. 2 was repeated with the dynamic equations replaced by a Lotka-Volterra model with high-order interactions: $\dot{x}_i = x_i\left(r_i + \sum_{j=1}^{N} A_{ij}x_j + \sum_{j=1}^{N}\sum_{k=1}^{N} B_{ijk}x_jx_k + \sum_{j=1}^{N}\sum_{k=1}^{N}\sum_{l=1}^{N} C_{ijkl}x_jx_kx_l + \cdots\right)$, with the interaction matrices *A,B,C* defined in the same manner as in Fig. 2, and the growth rates r_i set to 1/*N*. This scaling of r_i with *N* is needed to avoid systematic scaling of the total species abundance with the number of species. Still, this model is not strictly normalized, so cases where the total species abundance diverged appear. These diverging cases were also counted as unfeasible (defined by total abundance > 100**N*). Resulting slopes: -1.24 ± 0.21 for pairwise interactions (red), 0.13 ± 0.15 for 3-way interactions (blue) and 1.26 ± 0.14 for 4-way interactions (green).

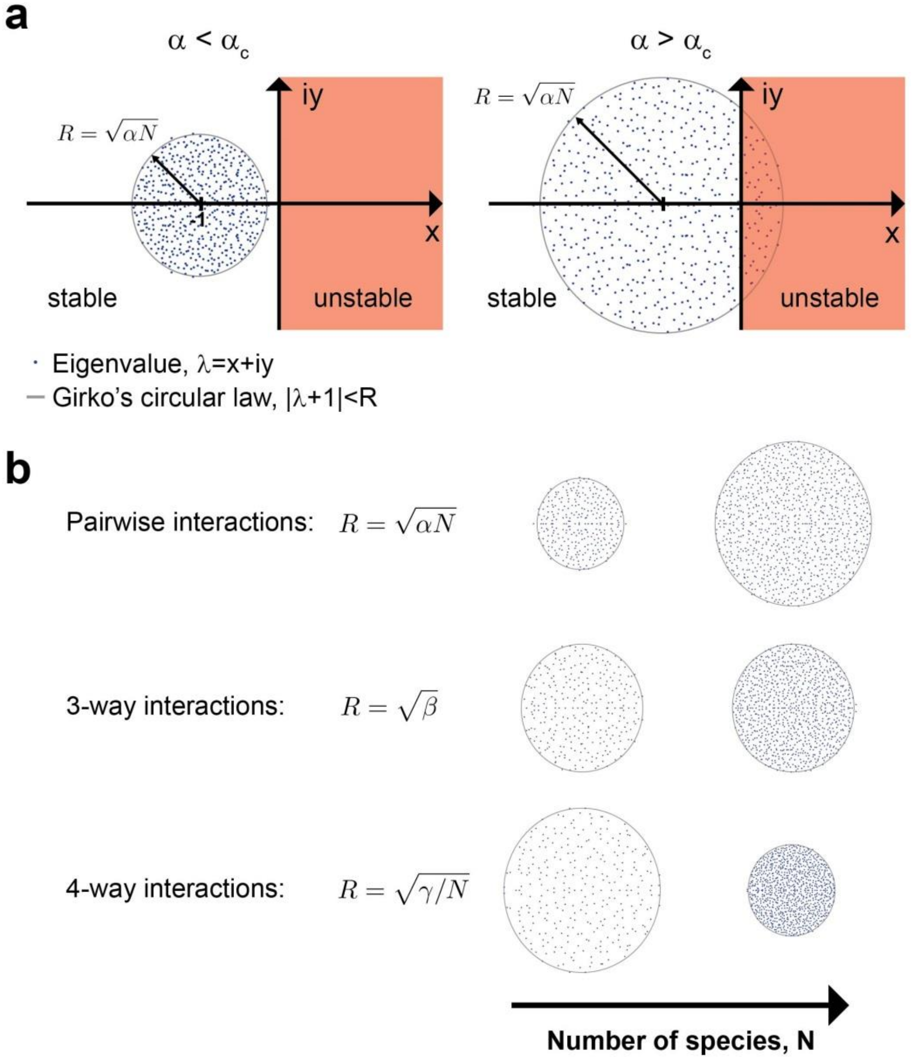

Supplementary Figure 6: Stability analysis in pairwise and high-order interactions through Girko's circular law. a, Deriving May's result: the eigenvalues of the pairwise interactions matrix $A = \sqrt{\alpha}\tilde{A} - I$ for a community consisting of $N = 500$ species with pairwise interaction strength below the critical threshold (left) and above the critical threshold (right). According to Girko's circular law, as $N \to \infty$ the distribution of the eigenvalues of A converges to a uniform distribution over a disc of radius $R = \sqrt{\alpha N}$ centered at (-1, 0). If $R < 1$ (left), this disc is included in the left half plane, so that all the eigenvalues have negative real parts, and the fixed point is stable; otherwise, the disc overlaps with the right half plane, and positive eigenvalues may occur. **b,** While the radius of the disc of eigenvalues of the effective pairwise interactions matrix A_{eff} increases with N for pairwise interactions, it is independent on N in the case of 3-way interactions, and inversely proportional to N when interactions are 4-way.

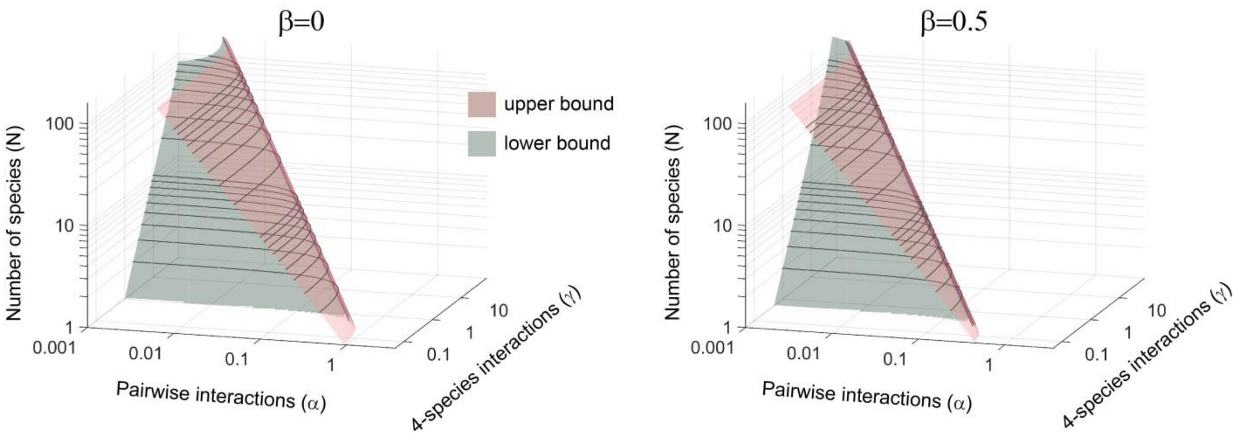

Supplementary Figure 7: Lower and upper bounds on diversity over a range of pairwise and 4-way interaction strengths. The lower and upper bound on diversity, defined by the roots of Eq. 2, are given as a function of α and γ, without 3-way interactions (left, β=0) and with 3-way interactions (right, β=0.5). While increasing the pairwise interactions decreases the upper bound on the range of stable diversities, an analogous lower bound increases with 4-way interactions. Introducing 3-way interactions narrows the range of stable diversities for given strengths of pairwise and 4-way interactions.

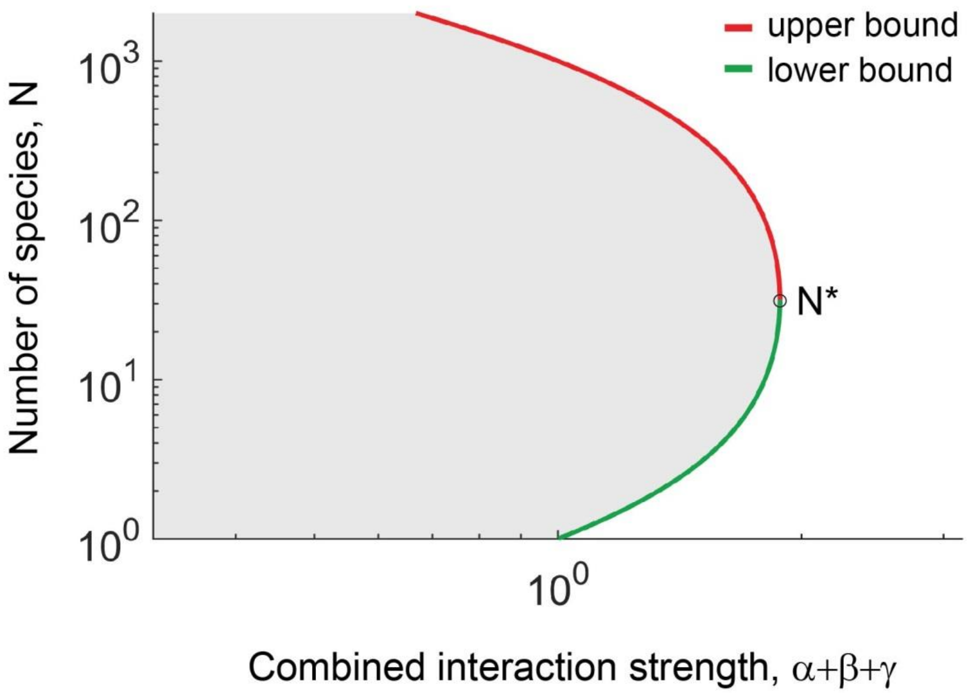

Supplementary Figure 8: Range of stable diversities over combined pairwise, 3-way and 4-way interaction strength. As the total interaction strength is increased, the lower and upper bounds on the number of species grow closer until they narrow around a defined number of species N^*. Here, 3-way interactions were added to the setting of Fig. 4 while keeping the relative strengths of pairwise and high-order interactions fixed ($\beta = \gamma = 10^3\alpha$). 3-way interactions affect the values of the lower and upper bounds and of the combined interaction strength where the two bounds meet, but do not change the qualitative behavior.

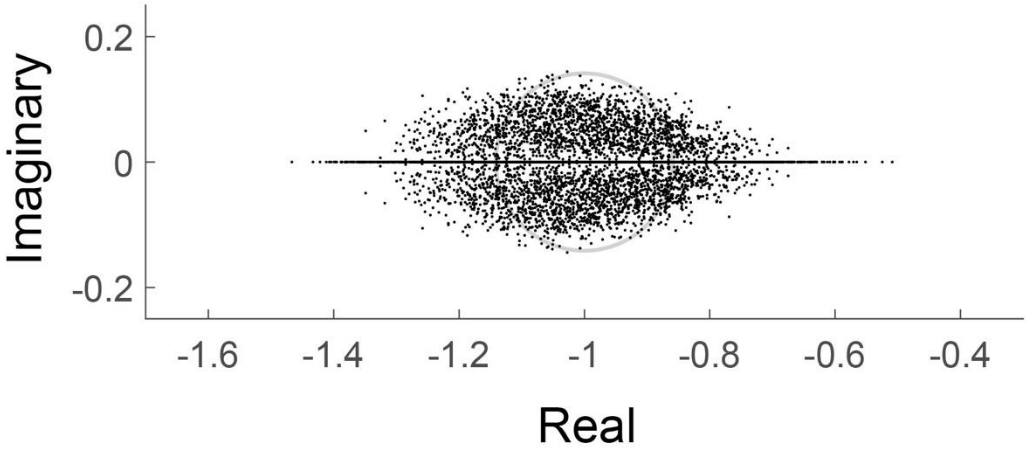

Supplementary Figure 9: Example for eigenvalue distribution of actual Jacobians at steady state. The Jacobians of 300 simulations with pairwise interactions only $\alpha = 10^{-3}$ and *N*=20 species were calculated at the end of the simulation. Their eigenvalues times *N* are shown (black dots), compared to the circle (gray line) of radius $\sqrt{\alpha N}$ around $(-1, 0)$ where the eigenvalues of our approximation to the Jacobian A^{eff} are uniformly distributed. Eigenvalues corresponding to the eigenvector associated with the normalization were excluded.
